# Benefits of dexmedetomidine during noninvasive mechanical ventilation in major abdominal surgery patients with postoperative respiratory failure

**DOI:** 10.3389/fsurg.2024.1357492

**Published:** 2024-05-09

**Authors:** Fatma Yildirim, Irem Karaman, Mehmet Yıldırım, Harun Karabacak

**Affiliations:** ^1^ General Surgery Intensive Care Unit, Department of Pulmonary and Critical Care Medicine, Dışkapı Yıldırım Beyazıt Research and Education Hospital, University of Health Sciences, Ankara, Türkiye; ^2^ School of Medicine, Bahçeşehir University, Istanbul, Türkiye; ^3^ Department of Internal Medicine - Internal Medicine Intensive Care Unit, Dışkapı Yıldırım Beyazıt Research and Education Hospital, University of Health Sciences, Ankara, Türkiye; ^4^ Department of General Surgery, Dışkapı Yıldırım Beyazıt Research and Education Hospital, University of Health Sciences, Ankara, Türkiye

**Keywords:** general surgery, postoperative pulmonary atelectasis, non invasive ventilation, dexmedetomidine, respiratory failure

## Abstract

**Objective:**

The efficacy of non-invasive mechanical ventilation (NIMV) on the postoperative ARF is conflicting and the failure rate of NIMV in this patient population is high. In our study, we hypothesized that the use of dexmedetomidine during NIMV in major abdominal surgical patients can reduce NIMV failure without significant side affect.

**Methods:**

Medical records of patients who underwent major abdominal surgery, admitted to our general surgery intensive care unit (ICU), developed postoperative ARF, received NIMV (with oro-nasal mask) and dexmedetomidine infusion were enrolled in this study. The infusion rate was adjusted to maintain a target sedation level of a Richmond Agitation-Sedation Scale (RASS) (−2)–(−3). The sedation was stopped when NIMV was discontinued.

**Results:**

A total of 60 patients, 42 (70.0%) male, and 18 (30.0%) female, with a mean age of 68 ± 11 years were included in the study. The mean APACHE II score was 20 ± 6. Dexmedetomidine was infused for a median of 25 h (loading dose of 0.2 mcg/kg for 10 min, maintained at 0.2–0.7 mcg/kg/h, titrated every 30 min). RASS score of all study group significantly improved at the 2 h of dexmedetomidine initiation (+3 vs. −2, *p* = 0.01). A targeted sedation level was achieved in 92.5% of patients. Six (10.0%) patients developed bradycardia and 5 (8.3%) patients had hypotension. The mean NIMV application time was 23.4 ± 6.1 h. Seven (11.6%) patients experienced NIMV failure, all due to worsening pulmonary conditions, and required intubation and invasive ventilation. Fifty-three (88.3%) patients were successfully weaned from NIMV with dexmedetomidine sedation and discharged from ICU. The duration of NIMV application and ICU stay was shorter in NIMV succeded group (21.4 ± 3.2 vs. 29.9 ± 6.4; *p* = 0.012).

**Conclusion:**

Our study suggests that dexmedetomidine demonstrates effective sedation in patients with postoperative ARF during NIMV application after abdominal surgery. Dexmedetomidine can be considered safe and capable of improving NIMV success.

## Introduction

Anesthesia, postoperative pain, and surgery may induce respiratory modifications following major abdominal surgery. Thus, hypoxemia commonly develop in postoperative patients. Hypoxemia, decrease in pulmonary volumes and atelectasis can be associated with restrictive respiratory patterns and diaphragmatic dysfunction. Maintenance of adequate oxygenation in the postoperative period has major importance, particularly when pulmonary complications such as acute respiratory failure (ARF) occur. Non-invasive mechanical ventilation (NIMV) is increasingly used for the treatment of ARF in postoperative patients (1–3). Although routine or prophylactic use of NIMV is not recommended in patients with major abdominal surgery, some studies have shown that NIMV with both continuous airway pressure and bilevel positive airway pressure reduces atelectasis and decreases the risk of pneumonia more effectively than standard oxygen therapy after abdominal surgery (4, 5). These studies support the use of NIMV in the postoperative setting. However, the efficiency of NIMV on the post-operative ARF is conflicting and the failure rate of NIMV in these patient populations is high (6, 7). Agitation is an important factor and it is associated with the failure of NIMV (8). Pain due to drains, tubes, and surgery itself can cause agitation in patients with major abdominal surgery followed in the intensive care unit (ICU), and they all together can cause the failure of NIMV. Sedation during NIMV has been on the agenda in recent years in order to reduce the agitation of patients and to prevent NIMV failure that may occur due to agitation (9, 10).

Dexmedetomidine is a selective alpha-2 receptor agonist that possesses both sedative and analgesic properties with minimal respiratory depression (11). Dexmedetomidine was found preferable to benzodiazepine sedatives in critically ill, mechanically ventilated patients in the most recent guidelines (12, 13). While a few randomized studies have explored the effects of dexmedetomidine in non-surgical patients managed with NIMV, none of them have studied its impact in patients with major abdominal surgery (14, 15).

In this study, patients who had major abdominal surgery and developed hypoxic ARF while being followed in the ICU in the postoperative period and were treated with NIMV and dexmedetomidine were investigated in terms of NIMV success rate, duration of administration, complications and adverse events, and factors determining the success rate of NIMV. We hypothesized that dexmedetomidine can be used safely and may reduce NIMV failure in patients who underwent major abdominal surgery and required NIMV due to postoperative respiratory failure, and in patients at risk of NIMV failure due to agitation, and that dexmedetomidine could be used safely.

## Material and methods

In this observational study patients who underwent major abdominal surgery (gastrectomy, colorectal surgery, liver surgery, and pancreatectomy) and who were admitted to our general surgery ICU, between January 2019 and March 2020 were included. Patients receiving NIMV due to postoperative respiratory failure [an acute episode of hypoxemic ARF (PaO_2 _< 55 mmHg)], patients who had Richmond Agitation-Sedation Scale (RASS) ≥ + 2 and at risk of NIMV failure due to agitation and who were given dexmedetomidine for sedation were included.

The prospectively collected and recorded data were evaluated retrospectively. Sedation and NIMV applied according to standardized NIMV and medical treatment ICU protocols. Inclusion criteria: Patients who received NIMV as the first treatment modality for ARF, and who received dexmedetomidine infusion for longer than 2 h, and patients who were too agitated (RASS > + 2) to allow administration and maintenance of NIMV.

Exclusion criteria: Patients who were younger than 18 years of age, who had chronic obstructive lung disease (COPD), asthma, or other obstructive lung diseases such as destroyed lung, bronchiectasis, or chronic respiratory failure prior to operation, patients with congestive heart failurewith an ejection fraction of less than 50% and at risk for cardiogenic pulmonary edema in the post-operative period, patients with a previous cerebrovascular accident, patients who hadhigh baseline PaCO_2_ levels (>55 mmHg), patients with obesity and body mass index over 30 kg/m^2^, and patients who needed emergent intubation.

**Figure 1:**
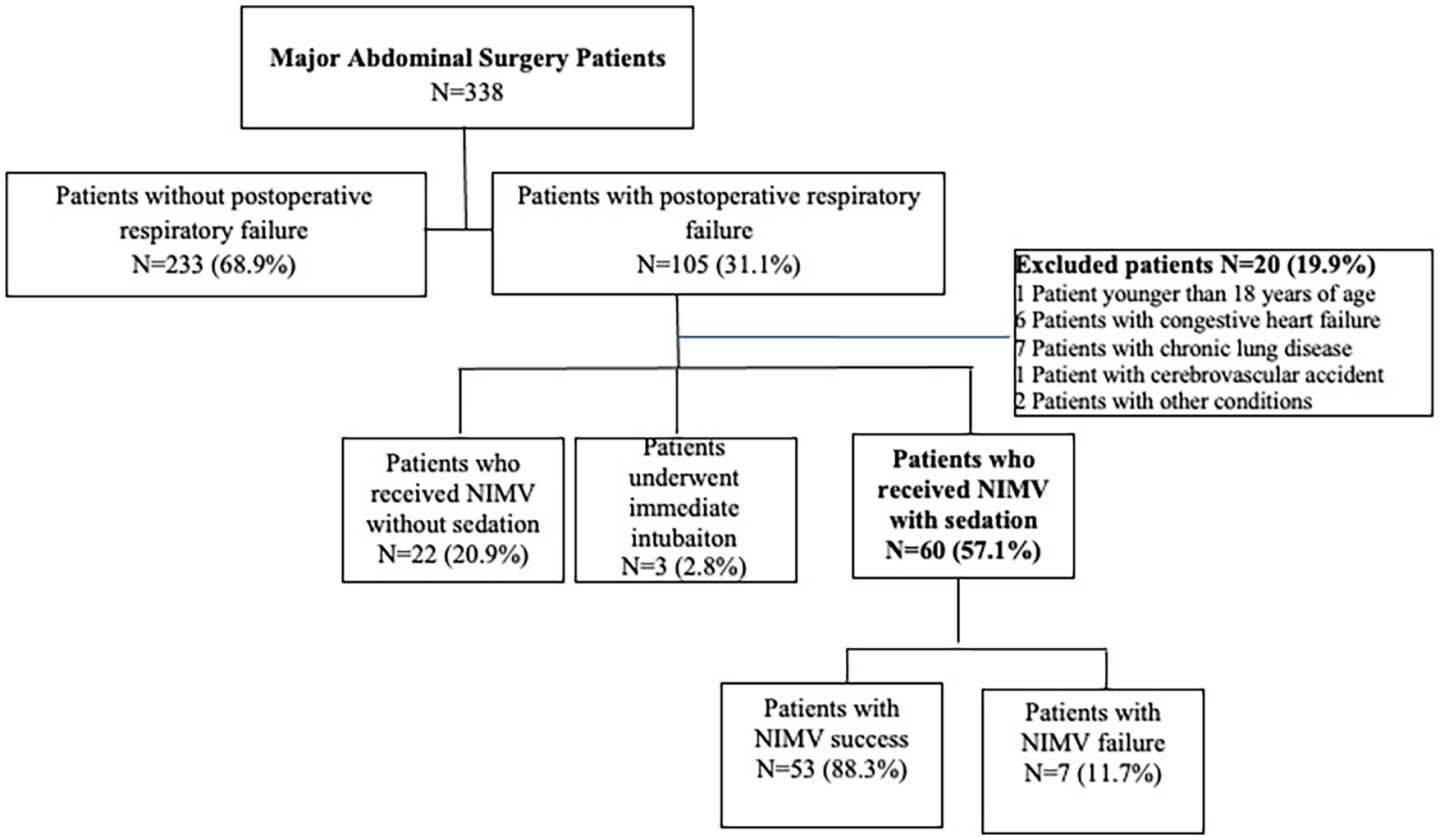
Flow chart of the study.

The flow chart of the study was shown in Figure 1.

## Non-invasive mechanical ventilation protocol

Indications for NIMV included hypoxemia (PaO_2_/FiO_2_ ratio of less than 200 mmHg while spontaneous breathing with supplemental oxygen by facemask), presence of severe dyspnea, and contraction of the accessory inspiratory muscles or paradoxical abdominal motion.

Non-invasive mechanical ventilation applied via oro-nasal mask with ICU mechanical ventilator (Puritan bennett 840®) in Spont® mode. Pressure support ventilation was increased by 2–3 cmH_2_O at 2–3 min intervals over the first 10–15 min according to the clinical response and tolerance of the patient to obtain an exhaled tidal volume of 6–8 ml/kg and a respiratory rate (RR) lower than 35 breaths/min. The positive end-expiratory pressure (PEEP) was repeatedly increased by 2 cmH_2_O to a maximum of 10 cmH_2_O until the FiO_2_ requirement became 65% or less to maintain the oxygen saturation (SpO_2_) above 92%. The ventilator settings were then adjusted on the basis of pulse oximetry and serial measurements of arterial blood gases. After this period, once the PEEP requirements decreased to 5 cmH_2_O, each patient was evaluated daily while breathing supplemental oxygen without ventilatory support for 15 min. NIMV and drug sedation were reduced progressively in accordance with the degree of clinical improvement and were discontinued if the patient stably maintained a respiratory rateof 15 breaths/minute and SpO_2_ above 92%.

Failure of NIMV was defined as the need for endotracheal intubation and invasive mechanical ventilation within 24 h of NIMV initiation. The decision to perform endotracheal intubation was made by the critical care specialist according to the usual criteria used in the unit: the inability to maintain a PaO_2_/FiO_2_ ratio >100 mmHg for 1 h of NIMV, cardiac arrest, respiratory arrest, respiratory pauses with loss of consciousness, severe encephalopathy, agitation not controlled by sedation, shock, or deterioration in gas exchange (PaCO_2 _> 55 mmHg) (15).

NIMV-related complications such as claustrophobia, oronasal dryness, excessive air leak, pain on the face due to NIMV mask, and other complications were recorded 48 h after NIMV discontinuation.

## Dexmedetomidine administration and adverse events

We applied intravenous (IV) dexmedetomidine according to our general surgery ICU protocol, which was a loading dose of 0.2 mcg/kg for 10 min, maintained at 0.7 mcg/kg/h, titrated every 30 min. The infusion rate was adjusted to maintain a target sedation level of a RASS score of (−2)–(−3). Sedation was stopped when NIMV was discontinued (16).

Adverse events were assessed and monitored by the responsible critical care spesialist and were recorded from the first dose of the study drug until 48 h after dexmedetomidine discontinuation. Our ICU protocol pre-specified that bradycardia and hypotension were considered adverse events if the systolic blood pressure was less than 80, diastolic blood pressure was less than 50, or the heart rate was less than 40 /min. A greater than 30% change from the baseline heart rate or blood pressure was also considered an adverse event. Other adverse events including nausea, vomiting, aspiration, and delirium were also recorded.

## Statistical analysis

All statistical analyses were performed using SPSS for Windows (Version 21.0, SPSS Inc.). We performed a power analysis to calculate the sample size, and 41 patients were found to be necessary for a significance level of 0.05. Nominal variables were expressed as mean ± SD (standard deviation) and processed as continuous variables. All tests were interpreted using a significance level of *p* < 0.05. Kolmogorov-Smirnov test was used to determine whether the data were distributed normally. Descriptive statistical methods (mean, standard deviation, median, frequency) were used when presenting the data.

## Results

Sixty patients, 42 (70.0%) male and 18 (30.0%) female, were included in the study. The mean age of the patients was 68 ± 11 years. The mean Acute Physiology and Chronic Health Assessment Score II (APACHE II) score was 20.0 ± 6.0, and American Society of Anesthesiologists (ASA) score was 3 ± 1. The most common comorbidities were hypertension (41.7%) and diabetes mellitus (33.3%). The mean Charlson Comorbidity Index was 3 ± 1; 93.3% of the patients had undergone elective surgery with gastrointestinal system malignancy. The most common surgeries were colorectal surgery (45.0%) and gastric surgery (36.7%). The mean RASS score of the patients at the beginning of NIMV was +3 ± 1, the mean respiratory rate was 27.5 ± 3.5 breaths/minute, and the mean PaO_2_ was 50.1 ± 4.5 mmHg. Other characteristics of the patients and hemodynamic parameters at the beginning of NIMV and the results of arterial blood gas analysis were shown in detail in Table 1.

**Table 1:** General characteristics, types of surgery, arterial blood gas analysis, hemodynamic parameters and non-invasive application time of all study group.

Characteristics	*N* = 60 (%)
Age (mean ± SD)(years)	68 ± 11
Gender (Male/Female)	42/18 (70/30)
APACHE II (mean ± SD)	20.0 ± 6.0
ASA (mean ± SD)	3 ± 1
Charlson comorbidity index (mean ± SD)	3 ± 1
Comorbidities	
Atherosclerotic heart disease	15 (25.0)
Hipertension	25 (41.7)
Diabetes mellitus	20 (33.3)
Aritmia	5 (8.3)
Gastrointestinal system malignancy	56 (93.3)
RASS score at the NIMV initiation	+3 ± 1
Type of surgery	
Gastrectomy	22 (36.7)
Colorectal surgery	27 (45.0)
Liver surgery	4 (6.7)
Pancreatectomy	7 (11.7)
Hemodynamic parameters at NIMV Initiation	
Respiratory rate (breaths/minute)	27.5 ± 3.5
Heart rate (beats/min)	114 ± 8
Systolic blood pressure (mmHg)	102 ± 28
Sp0_2_ (%)	85.2 ± 3.1
Blood gas analysis at NIMV Initiation	
pH	7.35 ± 0.02
PaO_2_ (mmHg)	55.1 ± 4.5
PaCO_2_ (mmHg)	43.2 ± 2.3
SpO_2_ (%)	87.0 ± 2.5
NIMV application time (hour)(mean ± SD)	23.4 ± 6.1

APACHE II, acute physiology and chronic health assessment score II; ASA, American Society of Anesthesiologists Score; RASS, richmond agitation-sedation scale; SD, standart deviation; NIMV, non-invasive mechanical ventilation.

Serially RASS scores were evaluated during the NIMV application. The RASS score of the study group significantly improved after one hour of NIMV with dexmedetomidine initiation (+3 vs. −2, *p* = 0.01). The targeted sedation level was achieved in 92.5% of patients.

Dexmedetomidine was infused for a median of 25 h with a median hourly dose across the patient cohort of 0.2 mcg/kg/h (range, 0.2–0.7 mcg/kg/h).Six (10.0%) patients developed bradycardia (30% change from the baseline heart rate) and 5 (8.3%) patients had hypotension (30% change from the baseline blood pressure). None of the patients who experienced hypotension or bradycardia required intervention. None of the patients experienced delirium or withdrawal.

Complications related to NIMV application were encountered in 7 (11.7%) patients (2 claustrophobia, 2 oronasal dryness, 1 excessive air leak, 2 pain on the face due to mask). None of these complications resulted in the discontinuation of NIMV. Complications were resolved by adjusting the NIMV humidifier and loosening and replacing the mask (Table 2).

**Table 2:** Complications observed with Non-invasive mechanical ventilation and adverse events related to dexmedetomidine.

Characteristics	*N* = 60 (%)
Complications related with NIMV	
Claustrophobia	2 (3.3)
Oronasal dryness	2 (3.3)
Excessive air leak	1 (1.7)
Pain on the face due to mask	2 (3.3)
Adverse event-related with dexmedetomidine	
Cardiovascular	
Bradycardia	6 (10.0)
Bradycardia requiring intervention	0 (0)
Hypotension	5 (8.3)
Hypotension requiring intervention	0 (0)
Delirium/withdrawal	0 (0)
Nausea/vomiting	0 (0)
Aspiration	0 (0)
Respiratory tract infection	0 (0)

NIMV, non-invasive mechanical ventilation.

Seven (11.6%) patients experienced NIMV failure, all due to worsening pulmonary conditions, and required intubation and invasive ventilation. Fifty-three (88.3%) patients were successfully weaned from NIMV under dexmedetomidine sedation and discharged from ICU. The group that experienced NIMV failure was older (69.3 ± 8.0 vs. 59.5 ± 5.0 years; *p* = 0.024) and had more tachypnea at the start of NIMV (27.5 ± 3.5 vs. 21.7 ± 2.7 breaths/min; *p* = 0.046) and had a lower PaO_2_/FIO_2_ ratio (158.1 ± 30.1 vs. 164.7 ± 24.2 mmHg, *p* = 0.031). NIMV application time and ICU stay were shorter in NIMV succeded group [(21.0 ± 3.2 vs. 29.9 ± 6.4; *p* = 0.019), (4.5 ± 1.5 vs. 2.5 ± 0.5 days; *p* = 0.042) respectively]. There was no difference between the two groups in terms of other features, hemodynamic findings, and other arterial blood gas findings. At the end of the 12th hour, an improvement in hypoxemia and an increase in the PaO_2_/FIO_2_ ratio were observed in both groups (Table 3).

**Table 3:** Comparison of characteristics of Non-invasive failure group and successful group.

Characteristics	NIMV failure group (*N* = 7, %)	NIMV successful group (*N* = 53, %)	*p*
Age (mean ± SD)(years)	69.3 ± 8.0	59.5 ± 5.0	**0**.**024**
Gender (Male/Female)	5/2 (71.4/28.6)	7/28 (20/80)	0.301
APACHE II (mean ± SD)	17 ± 6	14 ± 6	0.214
Charson comorbidity indeksi	3 ± 1	2 ± 0	0.652
Basal hemodynamic values at NIMV initiaiton			
Heart rate (beats/min)	101 ± 14	97 ± 11	0.601
Respiratory rate (breaths/min)	27.5 ± 3.5	21.7 ± 2.7	**0**.**046**
Systolic blood pressure (mmHg)	132 ± 17	133 ± 19	0.623
Body temperature (°C)	36.4 ± 0.1	36.5 ± 0.1	0.831
Blood gas analysis at NIMV Initiaiton			
pH	7.35 ± 0.04	7.38 ± 0.02	0.340
PaO_2_ (mmHg)	55.1 ± 3.0	56.4 ± 2.5	0.649
PaCO_2_ (mmHg)	42.2 ± 2.0	41.2 ± 0.9	0.480
SpO_2_ (%)	87 ± 2	88 ± 1	0.312
PaO_2_/FIO_2_(mmHg)	158.1 ± 30.1	164.7 ± 24.2	**0**.**031**
PaO_2_/FIO_2_ at the 1st hour of NIMV	178.9 ± 28.2	180.1 ± 19.3	0.773
PaO_2_/FIO_2_ at the 12 h of NIMV	208.1 ± 10.1	223.5 ± 2.3	0.293
NIMV Application time (hour)	29.9 ± 6.4	21.0 ± 3.2	**0**.**019**
ICU stay (day)	4.5 ± 1.5	2.5 ± 0.5	**0**.**042**

APACHE II, acute physiology and chronic health assessment score II; NIMV, non-invasive mechanical ventilation; ICU, intensive care unit.

*Bold values denote statistical significance at the *p* < 0.05 level.

Surgical site infection (SSI) is the most common postoperative after major abdominal surgery causing pain and suffering to patients. In our patients we detected SSIs in 3.0 (5%) patients. Postoperative sepsis after colorektal surgery is also another important complication. In our study only 1 (1.66) patient had intraabdominal sepsis. But none of them experienced NIMV failure.

## Discussion

In our study, initiating dexmedetomidine soon after NIMV initiation in agitated patients with major abdominal surgery both improved NIVM tolerance and helped to maintain sedation at the desired goal without significant side affect. Also it decreased the duration of NIMV application and shortened the length of stay in the ICU and the NIMV failure rate was 11.7%.

Hypoxemia develops in 30%–50% of patients after abdominal surgery and it can be well-tolerated in some patients without any symptoms (17). However, hypoxemia can also progress to severe ARF. The etiology for the development of hypoxemic acute respiratory failure postoperatively is multifactorial and partly related to atelectasis due to hypoventilation and collapsed alveoli, retained secretions, and diaphragmatic dysfunction. NIMV can reverse the loss of pulmonary volume by increasing lung ventilation, reopening atelectatic alveoli, and improving gas exchange (18). The residual effect of general anesthesia and discomfort and pain caused by the drainsmay causeagitation in post-operative surgical patients. These conditions pose a risk of NIMV failure. In recent years, the use of dexmedetomidine has become widespread in ICUs for sedation (19). The use of dexmedetomidine during NIMV administration in surgical patientsundergoing major abdominal surgerywith postoperative respiratory failure has not been studied extensively in the literature.

In the study conducted by Şenoğlu et al. (20) from Turkey, midazolam and dexmedetomidine were compared in terms of the level of sedation they provided in patients with acute exacerbation of COPD and in need of NIMV. Sedation level was followed by Ramsay Sedation Scale, Riker Sedation-Agitation Scale as well as Bispectral Index. In this study, which aimed at a sedation level similar to our study, they showed that dexmedetomidine is as effective as midazolam in providing effective sedation during NIMV and less dose adjustment is required to maintain the sedation level (20). In our study sedation target achieved in 92.5% of patients with dexmedetomidine.

In our study, NIMV failure and intubation were observed in 11.7% of the patients, but no mortality was encountered in the follow-up patients. All patients were weaned, exubated and discharged from ICU. The age, the respiratory rate at the beginning of NIMV, and the degree of hypoxemia of patients with NIMV failure were higher, and the improvement in hemodynamic parameters in this group was less in the 1st hour of NIMV. All these factors are identified as risk factors for NIMV failure, which have been previously noted in many studies (8). However, no difference was observed between the two groups in terms of APACHE II score, comorbidity (Charlson comorbidity index), and ASA score, which were reported as significant risk factors for NIMV failure in previous studies. This was attributed to the fact that our study group was more homogeneous and consisted of only surgical patients with post-operative respiratory failure.

In the study of Devlin et al. (21) in which the same dexmedetomidine doses (loading dose 0.2 mcg/kg for 10 min, titrated every 30 min maintained at 0.7 mcg/kg/h) were used as our study, patients with ARF who were treated with NIMV within 8 h were randomized to receive IV dexmedetomidine or placebo. The sedation level was determined on Sedation-Agitation Scale 3–4. In this study, early use of dexmedetomidine did not increase tolerance to NIMV, on the contrary, the use of dexmedetomidine prolonged the duration of NIMV and caused deeper sedation (21). However, in this study, patients with pain and agitation were additionally given midazolam or fentanyl. Both agents are agents that can deepen sedation and cause respiratory depression in patients. In our study, in addition to dexmedetomidine, only paracetamol was used as a pain reliever. In addition, our patient group consisted of post-operative patients, and the PaO_2_/FIO_2_ ratios of our patients were higher than the patients in Devlin et al's study.

In the study of Xie et al. (22) in which they compared dexmedetomidine and midazolam during NIMV in COVID-19 patients followed up in the ICU, Ramsay agitation score, hemodynamic parameters, and arterial blood gas results were evaluated. Mean blood pressure and heart rate were decreased in the sedated patients, with more decrement observed in the dexmedetomidine group.In the dexmedetomidine group, bradycardia was encountered in 2 patients, and NIMV was unsuccessful in 1 patient. In this study, in which the Ramsay score was kept between 2 and 3, they stated that appropriate sedation could increase the effectiveness of NIMV. In the same study, while PaO_2_ pressure increased in the groups receiving dexmedetomidine and midazolam, it did not increase in the control group which was not given sedation (79.0 ± 6.5 vs. 79.0 ± 8.9 vs. 70.0 ± 7.8; *p* < 0.005) (20). In our study, bradycardia was encountered in 6 patients and NIMV failure was encountered in 7 patients, which we attributed to the higher number of patients included in our study. In our study, the PaO_2_ values of the patients increased in the 1st and 12th hours in NIMV successed group.

A randomized crossover study by Deletombe et al. (10) investigated the use of dexmedetomidine during NIMV in patients with blunt chest trauma, and dexmedetomidine was started 1 h before NIMV. The RASS score was lower in the dexmedetomidine group [−0.8 (−1.0:0.0) vs. 0.0 (−0.5:0.0); *p* < 0.01]. In our study, RASS scores improved in both the NIMV successful group and the failure group.

In our study, complications related to NIMV were encountered in 7 (11.7%) patients, however, these complications were mild and did not cause NIMV arrest in any of the patients. According to the literature, such non-serious complications may be encountered in patients who underwent NIMV with an oro-nasal mask (1). These minor complications can be prevented by choosing the appropriate mask size for the patient's face and using a heater-humidifier. The most serious complication during NIMV is the development of cardiac arrest (23). We did not encounter this serious complication in any of our patients in our study.

Another common postoperative complication following colorectal procedure is the surgical site infections (SSI). SSIs have many negative consequences for patients (24). In our study we faced 5% SSIs and 1.66% intraabdominal sepsis. Mulita et al. Investigated the etiology as well as the risk factors associated with the development of postoperative sepsis (25). They diagnosed in 18 (12.77%) cases, with anastomotic leakage being the most frequent cause (3.55%). Postoperative sepsis was significantly more common among patients over 65 years old, ASA score >2, and also with associated comorbidities such as diabetes and cardiovascular disease (25).

This study had some limitations. First of all, it was a single-center study and the number of patients was limited. There was no comparison between the dexmedetomidine untreated group and there was no placebo-controlled group. However, as far as we know, this is the first study conducted by intensive care specialists on patients who underwent major abdominal surgery and developed postoperative respiratory failure in our country.

Our study suggests that dexmedetomidine demonstrates effective sedation in patients with postoperative ARF during NIMV application after abdominal surgery and it can be considered safe and capable of improving NIMV success. NIMV administration and IV dexmedetomidine application enable more effective use of surgical ICU beds and reduce the costs. However, if surgical patients who underwent NIMV and started IV dexmedetomidine are tachypneic and have severe hypoxemia, the risk of failure of NIMV should be considered and these patients should be followed more closely.

## Data Availability

The raw data supporting the conclusions of this article will be made available by the authors, without undue reservation.

## References

[1] Ozyılmaz E Kaya A. The effect of non-invasive mechanical ventilation in postoperative respiratory failure. Tuberk Toraks. (2012) 60(2):185–92. 10.5578/tt.2702 22779943

[2] Jaber S De Jong A Castagnoli A Futier E Chanques G. Non-invasive ventilation after surgery. Ann Fr Anesth Reanim. (2014) 33(7–8):487–91. 10.1016/j.annfar.2014.07.742 25168304

[3] Squadrone V Coha M Ceruti E Schellino MM Biolino P Occella P Continuous positive airway pressure for treatment of postoperative hypoxemia. JAMA. (2005) 293:589–95. 10.1001/jama.293.5.589 15687314

[4] PRISM trial group. Postoperative continuous positive airway pressure to prevent pneumonia, re-intubation, and death after major abdominal surgery (PRISM): a multicentre, open-label, randomised, phase 3 trial. Lancet Respir Med. (2021) 9(11):1221–30. 10.1016/S2213-2600(21)00089-8 34153272

[5] Ferreyra GP Baussano I Squadrone V Richiardi L Marchiaro G Del Sorbo L Continuous positive airway pressure for treatment of respiratory complications after abdominal surgery: a systematic review and meta-analysis. Ann Surg. (2008) 247(4):617–26. 10.1097/SLA.0b013e3181675829 18362624

[6] Jaber S Delay JM Chanques G Sebbane M Jacquet E Souche B Outcomes of patients with acute respiratory failure after abdominal surgery treated with noninvasive positive pressure ventilation. Chest. (2005) 128:2688–95. 10.1378/chest.128.4.2688 16236943

[7] Redondo FJ Madrazo M Gilsanz F Uña R Villazala R Bernal G. Helmet noninvasive mechanical ventilation in patients with acute postoperative respiratory failure. Respir Care. (2012) 57:743–52. 10.4187/respcare.01170 22152725

[8] Ozyilmaz E Ugurlu AO Nava S. Timing of noninvasive ventilation failure: causes, risk factors, and potential remedies. BMC Pulm Med. (2014) 14(19):1–10. 10.1186/1471-2466-14-19 24520952 PMC3925956

[9] Yıldırım F Kara İ Ortaç Ersoy E. Sedation during noninvasive mechanical ventilation. Tuberk Toraks. (2016) 64(3):230–9. 10.5578/tt.10764 28366157

[10] Deletombe B Trouve-Buisson T Godon A Falcon D Giorgis-Allemand L Bouzat P Dexmedetomidine to facilitate non-invasive ventilation after blunt chest trauma: a randomised, double-blind, crossover, placebo-controlled pilot study. Anaesth Crit Care Pain Med. (2019) 38(5):477–83. 10.1016/j.accpm.2019.06.012 31319192

[11] Smuszkiewicz P Wiczling P Ber J Warzybok J Małkiewicz T Matysiak J Pharmacokinetics of dexmedetomidine during analgosedation in ICU patients. J Pharmacokinet Pharmacodyn. (2018) 45(2):277–84. 10.1007/s10928-017-9564-7 29290034 PMC5845053

[12] Romagnoli S Amigoni A Blangetti I Casella G Chelazzi C Forfori F Light sedation with dexmedetomidine: a practical approach for the intensivist in different ICU patients. Minerva Anestesiol. (2018) 84:731–46. 10.23736/S0375-9393.18.12350-9 29405671

[13] Devlin JW Skrobik Y Gelinas C Needham DM Slooter AJC Pandharipande PP Executive summary: clinical practice guidelines for the prevention and management of pain, agitation/sedation, delirium, immobility, and sleep disruption in adult patients in the ICU. Crit Care Med. (2018) 46:1532–48. 10.1097/CCM.0000000000003259 30113371

[14] Akada S Takeda S Yoshida Y Nakazato K Mori M Hongo T The efficacy of dexmedetomidine in patients with noninvasive ventilation: a preliminary study. Anesth Analg. (2008) 107(1):167–70. 10.1213/ane.0b013e3181732dc2 18635484

[15] Huang Z Chen YS Yang ZL Liu JY. Dexmedetomidine versus midazolam for the sedation of patients with non-invasive ventilation failure. Intern Med. (2012) 51:2299–305. 10.2169/internalmedicine.51.7810 22975538

[16] Sessler CN Gosnell MS Grap MJ Brophy GM O'Neal PV Keane KA The richmond agitation-sedation scale: validity and reliability in adult intensive care unit patients. Am J Respir Crit Care Med. (2002) 166(10):1338–44. 10.1164/rccm.2107138 12421743

[17] Liu K Scott JB Jing G Li J. Management of postoperative hypoxemia. Respir Care. (2021) 66(7):1136–49. 10.4187/respcare.08929 34006596

[18] Chiumello D Chevallard G Gregoretti C. Non-invasive ventilation in postoperative patients: a systematic review. Intensive Care Med. (2011) 37(6):918–29. 10.1007/s00134-011-2210-8 21424246

[19] Romera Ortega MA Chamorro Jambrina C Lipperheide Vallhonrat I. Fernández simón I indications of dexmedetomidine in the current sedoanalgesia tendencies in critical patients. Med Intensiva. (2014) 38(1):41–8. 10.1016/j.medin.2013.03.008 23683866

[20] Senoglu N Oksuz H Dogan Z Yildiz H Demirkiran H Ekerbicer H. Sedation during noninvasive mechanical ventilation with dexmedetomidine or midazolam: a randomized, double-blind, prospective study. Curr Ther Res Clin Exp. (2010) 71(3):141–53. 10.1016/j.curtheres.2010.06.003 24683260 PMC3967280

[21] Devlin JW Al-Qadheeb NS Chi A Roberts RJ Qawi I Garpestad E Efficacy and safety of early dexmedetomidine during noninvasive ventilation for patients with acute respiratory failure: a randomized, double-blind, placebo-controlled pilot study. Chest. (2014) 145(6):1204–12. 10.1378/chest.13-1448 24577019

[22] Xie W Zhong Z Li G Hou G Huang K Yu Z. A comparative study on clinical effects of dexmedetomidine and midazolam on patients with severe coronavirus disease 2019 on non-invasive ventilation. Zhonghua Wei Zhong Bing Ji Jiu Yi Xue. (2020) 32(6):677–80. 10.3760/cma.j.cn121430-20200305-00187 32684211

[23] Rolle A De Jong A Vidal E Molinari N Azoulay E Jaber S. Cardiac arrest and complications during non-invasive ventilation: a systematic review and meta-analysis with meta-regression. Intensive Care Med. (2022) 48(11):1513–24. 10.1007/s00134-022-06821-y 36112157 PMC9483519

[24] Panos G Mulita F Akinosoglou K Liolis E Kaplanis C Tchabashvili L Risk of surgical site infections after colorectal surgery and the most frequent pathogens isolated: a prospective single-centre observational study. Med Glas (Zenica). (2021) 18(2):438–43. 10.17392/1348-21 34080408

[25] Mulita F Liolis E Akinosoglou K Tchabashvili L Maroulis I Kaplanis C Postoperative sepsis after colorectal surgery: a prospective single-center observational study and review of the literature. Prz Gastroenterol. (2022) 17(1):47–51. 10.1007/s11377-021-00584-6 35371356 PMC8942007

